# *Strongyloides stercoralis* infection in San Marino Republic: first epidemiological data from an observational study

**DOI:** 10.1017/S0950268819000980

**Published:** 2019-05-29

**Authors:** E. D. Cappella, A. C. Piscaglia, A. Cadioli, S. Manoni, R. Silva, D. Buonfrate

**Affiliations:** 1 Clinical Pathology and Transfusion Medicine Unit – State Hospital, Istituto per la Sicurezza Sociale (ISS), Via Scialoja 20, 47893 Borgo Maggiore, Republic of San Marino; 2 Endoscopy and Gastroenterology Unit – State Hospital, Istituto per la Sicurezza Sociale (ISS), Via Scialoja 20, 47893 Borgo Maggiore, Republic of San Marino; 3 Department of Surgical Pathology and Cytology, Rimini Hospital – AUSL della Romagna, via Settembrini 2, 47900 Rimini, Italy; 4 Department of Infectious – Tropical Diseases and Microbiology, IRCCS Sacro Cuore Don Calabria Hospital, Via Sempreboni 5, 37024 Negrar, Verona, Italy

**Keywords:** Nematodes, parasitic disease epidemiology and control, strongyloidiasis

## Abstract

*Strongyloides stercoralis* is a neglected parasite that can cause death in immunocompromised individuals. There were no data on the epidemiology of *S. stercoralis* infection in San Marino Republic until two patients (one of whom died) were diagnosed with severe strongyloidiasis (hyperinfection) between September 2016 and March 2017. A serology test for *Strongyloides* spp. was introduced in routine practice in the laboratory of the State Hospital to test patients considered to be at risk for strongyloidiasis. Between August 2017 and August 2018, of 42 patients tested with serology, two (4.8%) were positive. An additional case was found by gastric biopsy. Two of the positive cases were presumably autochthonous infections (elderly people with no significant travel history), while the other was a probable imported case (young man born in Nigeria and settled in Europe since 2003). Epidemiology of strongyloidiasis in San Marino might be similar to Northern Italy, where a relevant proportion of cases was diagnosed in immigrants (mainly from sub-Saharan Africa) and in elderly Italians with eosinophilia. Screening for strongyloidiasis might be worthwhile in inhabitants of San Marino in the same categories of individuals, particularly those at risk of immune suppression.

## Introduction

*Strongyloides stercoralis* infection is a soil-transmitted helminthiasis, with an estimated global prevalence of about 350 million cases [1], mostly distributed in tropical and subtropical areas of the world, where sewage disposal is inadequate or absent, and human waste contaminates the environment [2]. In those settings, the larvae released with faeces have a free-living cycle in the soil, and in their infective stage they can infect humans by direct penetration of the skin. After migration in the organism involving the respiratory tract, the larvae mature into adult worms; while the male adults have never been retrieved from the human body, the female adults settle in the intestine. The newborn larvae hatch out from the eggs when still in the bowel and are released with faeces. However, some larvae mature into the infective stage before leaving the human body, and can re-infect the host by penetration of the perianal skin or the last part of the rectum. This is called ‘auto-infection’, and leads to the maintenance of the infection through the years, if not properly treated [2]. Strongyloidiasis is diagnosed in areas of temperate climate, as a result of both immigration from endemic countries and emergence of cases locally acquired in the past, when poor sanitation caused contamination of soil. In countries in the Mediterranean basin, autochthonous cases are mostly diagnosed in specific populations at risk of soil-transmitted infections, such as individuals who have been working as agricultural workers and/or have been living in rural areas in their youth [3, 4].

**Fig. 1.**
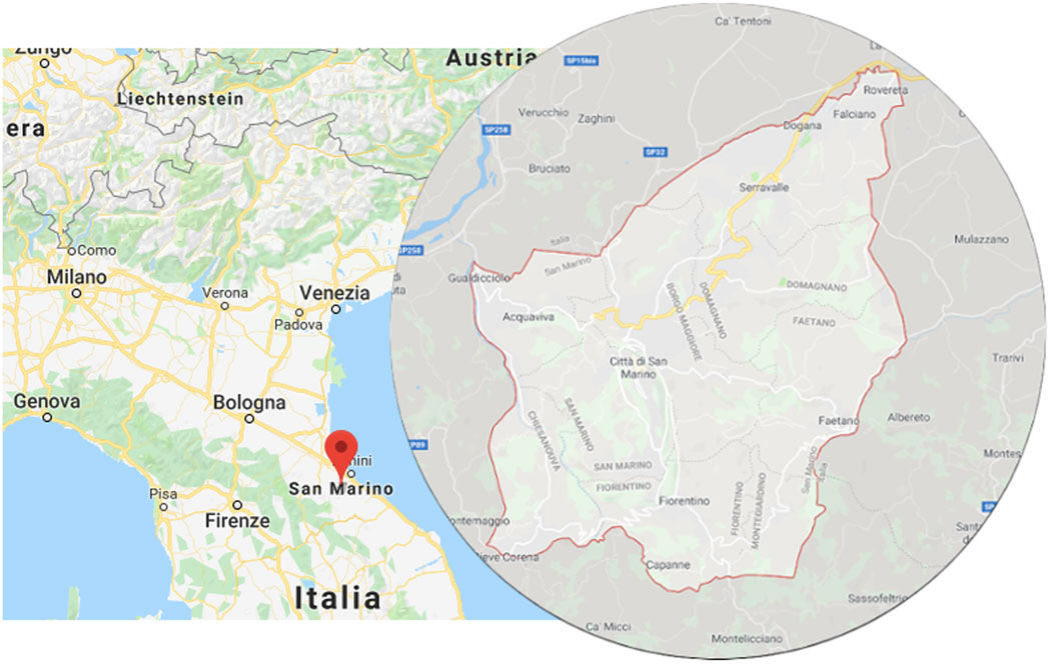
Map showing the geographical location of San Marino.

San Marino Republic is an independent state on the Adriatic side of central Italy (Fig. 1). The highest altitude of the country is 739 m above sea level, and most part of the Republic is hilly. The total resident population was estimated at 28 846 people in 2010, according to the latest census figures [5]. Residents with citizenship other than Italian/San Marino were 3.1%. Main characteristics regarding habits and sanitation (as well as language and currency) are shared with the surrounding Italian territory. Epidemiology of strongyloidiasis in the country was unknown until screening activities started in 2017, following the death of a patient from hyperinfection with no history of travel abroad. Previously, only sporadic cases of chronic strongyloidiasis had been found.

Aim of this work was to describe two cases of *S. stercoralis* hyperinfection diagnosed during a 6-month period in two patients admitted to the State Hospital in San Marino, and the results of following screening activities conducted over a 1-year period in patients at risk of infection.

## Methods

This was an observational, retrospective study. A physician working at the State Hospital in San Marino reviewed the patients' records to retrospectively collect the clinical and laboratory data. The data were collected and stored anonymously. The reported stool examinations were conducted, as for normal routine practice, at the microbiology laboratory of the State Hospital of San Marino. For the first patient, the serology was a *Strongyloides ratti* ELISA test (Bordier Affinity Products), while for the second patient the serology was an in-house immunofluorescence test (IFAT) [6]; both tests were carried out at the Department of Infectious – Tropical Diseases and Microbiology of IRCCS Sacro Cuore Don Calabria Hospital in Negrar, Verona, Italy. The latter department (referral centre for parasitology) also carried out polymerase chain reaction (PCR) for *S. stercoralis*. Histological examinations of biopsies were carried out at the Department of Surgical Pathology and Cytology of Rimini Hospital (Rimini, Italy).

Since August 2017, a commercial serology test (*Strongyloides* serology microwell ELISA, Scimedx Corporation) has been available at the microbiology laboratory of the State Hospital of San Marino for screening and individual diagnosis of patients referred by either general practitioners or specialists working in the hospital wards. We collected the results of the tests conducted between August 2017 and August 2018, and the main characteristics of the patients tested.

Demographic data were summarised using descriptive statistics and measures of spread to characterise the screened population. From contingency tables of test results and cross tables of symptoms, the proportion of patients with the condition and the main reasons of the patients' screening for *S. stercoralis* infection were assessed. Results were presented as proportions, median, plots and time to event plot, to visually examine the screening frequency within the study period.

## Results

### Case one

**Fig. 2.**
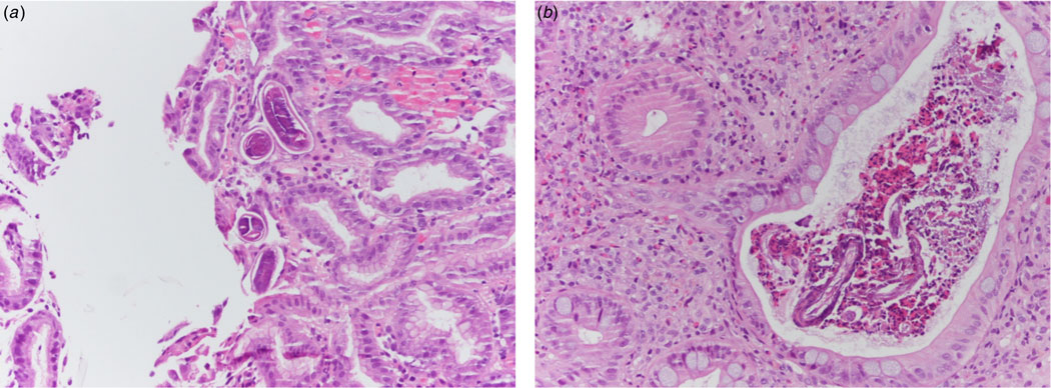
(a) Histological specimen from gastric biopsies of patient 1. Evidence of numerous *S. stercoralis* larvae in crypts (H&E 20× magnification). (b) Histological specimen from colonic biopsies of patient 2. Eosinophilic crypt abscess with evidence of *Strongyloides* larvae and eggs. The surrounding lamina propria shows a dense inflammatory infiltrate rich in eosinophils (H&E 20× magnification).

An 84-year-old patient with multiple chronic conditions (arterial hypertension, multifactorial anaemia, chronic obstructive pulmonary disease (COPD), prostatic hypertrophy, gout, diverticulosis of the colon, chronic renal failure) was admitted to the State Hospital in San Marino on the 9 August 2016 for diarrhoea and impaired general condition. Ten months before he had started taking prednisone 25 mg a day for worsening of the COPD, and a few days before admission he had undergone an oesophagogastroduodenoscopy (EGD) for epigastric pain, with evidence of diffuse oedema and hyperaemia of the gastric mucosa, and of an ulcerated polypoid lesion in the second portion of the duodenum. Upon admission, the full blood count showed mild normocytic anaemia (haemoglobin 10.8 g/dl), white blood cells and full blood count were within the normal range of values (in particular eosinophil count was 222 eosinophils/μl), C-reactive protein (CRP) was 5.20 mg/dl (normal values <1). The dose of diuretics already taken by the patient was increased and prednisone was continued; symptomatic treatment and cholestyramine were added. On the 8th day of hospitalisation an antibiotic treatment was started (piperacillin-tazobactam) and steroidal treatment was increased (prednisone was changed to parenteral methylprednisolone 20 mg twice a day) due to worsening respiratory conditions with signs of a pulmonary infiltrate at the chest X rays. However, the respiratory impairment continued worsening, and the patient started having nausea and vomiting with bile and blood. A total body computed-tomography (CT) scan showed diffused tree in bud sings, while excluding involvement of other organs. On the 17th day of hospitalisation, the results of the biopsies performed during EGD were communicated to the clinicians: nematodes compatible with *S. stercoralis* were present in the duodenal and gastric mucosa (Fig. 2a). In the meantime, *S. stercoralis* larvae were also found in the microscopic examination of a bronchoalveolar lavage. Microscopic examination of multiple stool and urine samples was negative. Treatment with albendazole 400 mg a day was hence started on the 26 August, and changed 3 days later with ivermectin 200 μg/kg/day following consultation with a tropical diseases specialist. Moreover, steroids were stopped. Nevertheless, the condition progressed to multi-organ failure and the patient died on the 2nd September. *Strongyloides* serology was positive, but the result was made available only 2 days after the death. Afterwards, previous clinical records were reviewed, showing that the patient had presented eosinophilia: 944 cells/μl in June 2016, 4490 cells/μl in August 2012.

### Case two

**Fig. 3.**
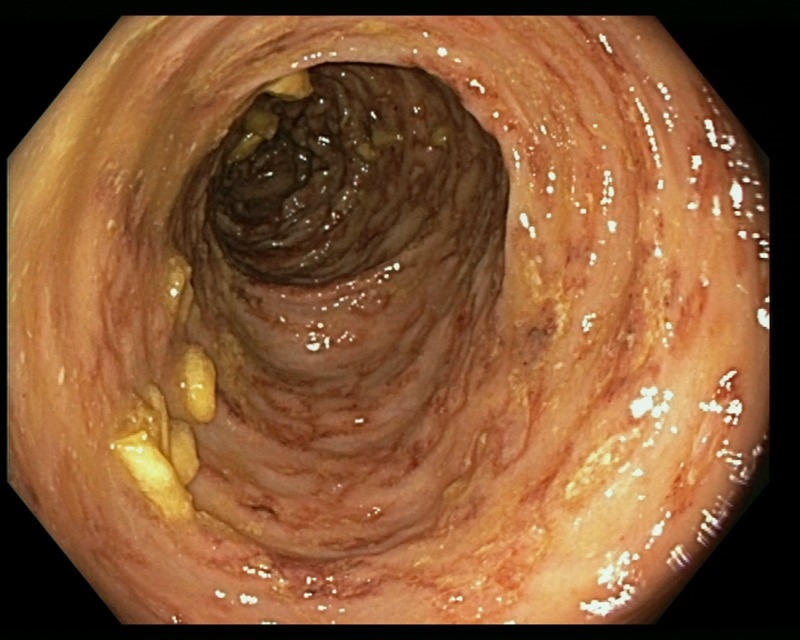
Erythematous and oedematous mucosa of the transverse colon, with aphtoid ulcers and petechiae.

An 85-year-old patient with moderate-severe chronic renal failure, insulin-dependent diabetes mellitus with peripheral polyneuropathy, COPD, prostatic hypertrophy and rheumatic polymyalgia with probable small-vessel vasculitis was admitted to the State Hospital in San Marino on the 8 March 2017 for fever, asthenia and hyporexia. Blood tests showed hypochromic microcytic anaemia (haemoglobin 9.2 g/dl), mild eosinophilia (558 eosinophils/μl), hyperglycaemia (265 mg/dl), increased erythrocyte sedimentation rate (99 mm/h, normal values <13) and CRP (8.43 mg/dl, normal values <1). Two sets of blood culture were negative. During the first days of hospitalisation, he was kept on his usual therapy, which included prednisone 12.5 mg/day. On the 13th March a contrast-enhanced CT scan was done, showing diffuse bowel and gastric wall thickening, thickening of the bronchial walls and pulmonary interstitial oedema. A colonoscopy demonstrated a flat lesion in the right colon, intestinal inflammation, ulcers and petechial spots (Fig. 3). The histological examination revealed lymphoplasmacellular and eosinophilic infiltration, epithelioid granuloma and multiple *S. stercoralis* larvae (Fig. 2b). Also, intranuclear inclusion bodies compatible with cytomegalovirus (CMV) infection were found; this infection was confirmed by PCR on blood, that showed 1888 UI/l viraemia. On the 16 March treatment with ivermectin 200 μg/kg/day and piperacillin/tazobactam 2.25 g three times a day (dosage adjustment for renal failure) was started. The dose of prednisone was gradually reduced. In the meantime, also stool examination, PCR and IFAT resulted in positive for *S. stercoralis*. Hence, on the 20 March, albendazole 400 mg twice a day was added. The general condition of the patient gradually improved, and CMV viraemia reduced spontaneously along with clinical recovery. Antihelminthic therapy was stopped 2 weeks after negativisation of stool microscopy, that was achieved after 21 days of treatment. The patient was discharged on the 7th April, in fair clinical condition. On the 30 May, he underwent a colonoscopy that showed almost complete resolution of the inflammation. The biopsy of the flat lesion (observed also in the previous examination) demonstrated a villous adenoma.

### Screening activities

Over the 1-year period, 43 patients were referred to the microbiology laboratory of the State Hospital in San Marino for ELISA testing. Twelve patients were referred from the Internal Medicine ward, one patient from the intensive care unit and 30 from the outpatient department. Median age of the patients tested was 66 years (interquartile range (IQR) 48–78), and 22 out of 43 (51.2%) were female. Thirty-four patients (79.1%) presented symptoms compatible with *S. stercoralis* infection; most symptoms involved the gastrointestinal tract (27 of 43 patients, 62.8% of patients tested), five patients had respiratory tract symptoms (11.6% of all patients tested), four patients presented weight loss (9.3%) and one patient reported itching. Median eosinophil value was 260 cells/μl (IQR 128–490). Reasons for the prescription of the test were: eosinophilia and symptoms compatible with *S. stercoralis* infection in nine cases; symptoms compatible with *S. stercoralis* infection (and no eosinophilia) in 25 cases; eosinophilia (with no symptoms) in five cases; the remaining four patients were tested in view of an immunosuppressant condition that could pose a *S. stercoralis*-infected patient at risk of hyperinfection.

**Fig. 4.**
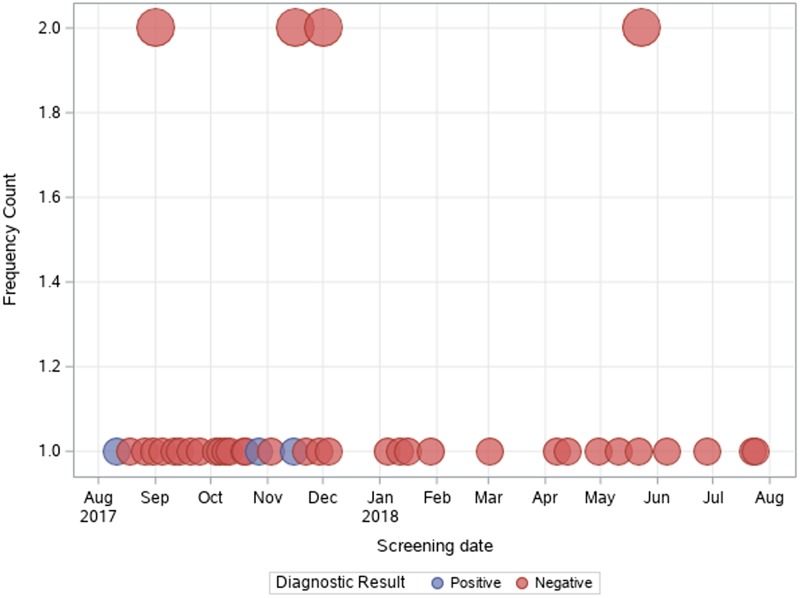
Number of tests (and results) performed per day over the study period.

**Fig. 5.**
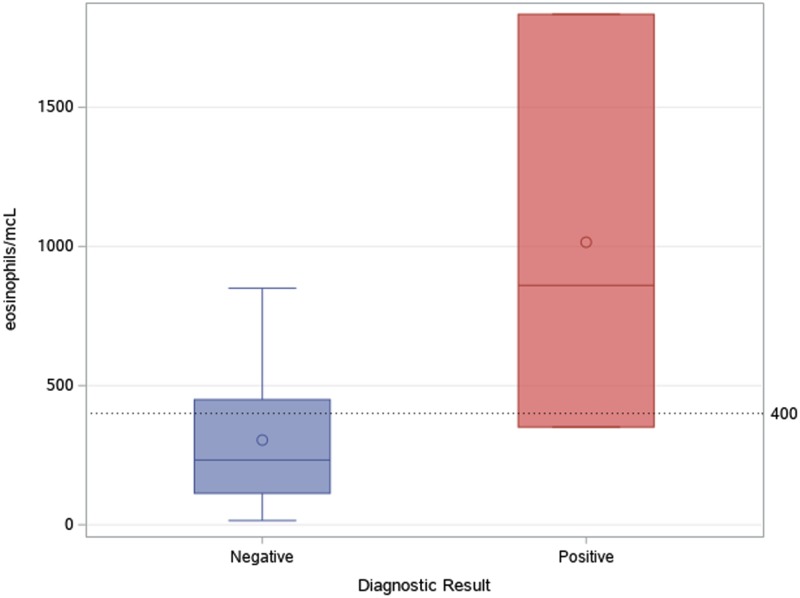
Median and IQR values of eosinophil count in patients with positive and negative serological result.

As shown in Figure 4, most tests were prescribed in the first semester of the activities (August 2017–February 2018): accounting for 74.4% of tests performed in the whole study period. Two patients out of 42 (4.8%) were found positive to serological screening (one autochthonous patient and one immigrant), but an additional autochthonous patient was diagnosed in the same study period by gastric biopsy. Two of the positive patients were born in San Marino, aged >80 years and had no history of travels outside San Marino/Italy (‘autochthonous cases’). The other positive patient was a 31-year-old man born in Nigeria and arrived in Europe (Italy first, then San Marino) in 2003. Median values of eosinophil count was 860 (IQR 351–1834) for positive patients, and 232 (IQR 113–449) for negative patients, as shown in Figure 5. Of note, the two autochthonous cases had moderate-high eosinophilia (from 860 to 1834 cells/μl upon diagnosis), while the patient from Nigeria had an eosinophil count of 351 cells/μl. All patients with positive serology presented compatible symptoms, in particular all patients reported abdominal pain and/or diarrhoea. The Nigerian patient had an obstruction of the bowel. One of them also reported weight loss and hyporexia. None of them was immunocompromised. The two patients from San Marino received treatment with ivermectin, with good clinical response. The African patient left San Marino before receiving treatment.

## Discussion

This work reports the first epidemiological data about strongyloidiasis in San Marino Republic. High prevalence of strongyloidiasis has been observed in the areas of higher altitudes [7], hence the transmission of the infection should have not been excluded in the hilly territory of San Marino. As the main characteristics of sanitation and population's habits are similar between Italy and San Marino, it was reasonable to presume that the epidemiology of *S. stercoralis* infection in San Marino could at least partially mirror what has been found in other areas of the Italian territory. In particular, in a previous work [4] conducted in three regions of Northern Italy, 8% of 1137 Italians over 60 years with eosinophilia resulted positive to a screening survey for strongyloidiasis. The different criteria used for the selection of the individuals tested in the two studies partially limit a comparison of the results, but also in this work all autochthonous cases were diagnosed in people over 70 years of age, including the two severe cases. The eosinophil count was normal in patient 1 at the time of hyperinfection. This is not unexpected, as a normal eosinophil count has been described in patients with complicated strongyloidiasis. Conversely, none of them reported itching and/or urticaria, the symptoms most frequently reported by positive patients in the previous study (and in many others [8]). The limited number of positive cases described here might be the cause of the discrepancy.

Most of the tests were carried out in the first 6 months of the study period, suggesting that the awareness of the health care givers was higher just after the serological test was made available, but then decreased. Specific protocols would be better implemented in the country, in order to guide local health care providers about patients who need testing, specifically when risk factors for strongyloidiasis are present (i.e. people older than 60 years who lived in rural areas in their youth, and/or with eosinophilia and/or compatible symptoms, or people at any age coming from endemic areas with eosinophilia and/or symptoms). For instance, the marked eosinophilia (4490 cells/μl) should have raised the index of suspicion for patient 1, who could have been saved if diagnosed and treated years before, or at least before the administration of steroids. The same can be said for patient 2, who had mild eosinophilia at the moment of diagnosis and fortunately survived. The African patient was diagnosed 14 years after his departure from Nigeria, where he had presumably acquired the infection in his childhood. This is possible because of the auto-infection cycle of *S. stercoralis*, and clinicians should be aware that immigrants can be at risk of the infection although they left endemic areas decades before.

Although none of the immunocompromised patients tested positive in this survey, it is worth to screen patients candidate to immunosuppression (when epidemiological risk of strongyloidiasis is present), in consideration of the risk of developing disseminated strongyloidiasis. Immunosuppressant factors include the use of steroids, that are frequently prescribed for many different conditions, and have been identified as triggers for the development of hyperinfection.

## Conclusions

The epidemiology of strongyloidiasis in San Marino Republic is probably similar to the one found in Northern Italy, with autochthonous cases diagnosed in individuals who acquired the infection in their youth (some decades ago, before sanitation conditions improved). Local protocols should be implemented to guide screening of patients at risk of infection.
